# Editorial: Innate Immunity in the Context of Osteoimmunology

**DOI:** 10.3389/fimmu.2020.00603

**Published:** 2020-04-07

**Authors:** Anita Ignatius, Cristina Sobacchi

**Affiliations:** ^1^Medical Center, Institute of Orthopedic Research and Biomechanics, Ulm University, Ulm, Germany; ^2^Milan Unit, CNR-IRGB, Milan, Italy; ^3^Humanitas Clinical and Research Center IRCCS, Rozzano, Italy

**Keywords:** osteoimmunology, innate immunity, fracture healing, bone regeneration, chemokines

The term “osteoimmunology” identifies the research field aimed at studying the crosstalk between cells of the skeletal and immune systems (1). The close relationship between these two systems is apparent based on the sharing of the same microenvironment (2), but it also extends beyond this through a network of signaling pathways and molecules acting in the pathophysiology of bone and immune cells (3). A large proportion of research in osteoimmunology has long focused on the effects elicited by adaptive immunity on bone, with rheumatoid arthritis as a prototypical disease condition (4). Only recently has the innate arm of the immune system received increasing attention in this framework (5). Indeed, osteonal macrophages, mast cells, and dendritic cells, in particular, have emerged as active players in skeletal remodeling and repair and in inflammation-induced bone loss (6–8). In parallel, novel concepts have been proposed regarding the capacity of bone cells to regulate immunity, suggesting, for example, the classification of osteoclasts as professional antigen-presenting cells and inflammatory osteoclasts as a different population compared to homeostatic osteoclasts (9, 10).

Hereby, the overall picture on the bone-immune system interplay gained additional complexity. This collection of articles reflects this topic and focusses on osteoimmunology with regard to innate immune cells/bone cells crosstalk.

## Macrophages and Bone Fracture Healing

Bone healing is a prototype for a regenerative process; indeed, at least ideally, the injured tissue undergoes a complete *restitutio ad integrum* without scar formation through the contribution of different cell types; in particular, this context offers the stage to many osteoimmunological interactions. In this issue, Stefanowski et al. investigated early events of vascularization at sites of bone regeneration in a murine osteotomy model. The authors showed *in vitro* and *in vivo* that the newly generated vessels, expressing markers of type H endothelium, transiently accumulated far from the fracture site, close to osteoprogenitors and macrophages. In particular, CX3CR1^+^F4/80^+^ cells were the most abundant macrophage population, having progressively infiltrated the hematoma prior to functional vascularization and persisted until remodeling. Overall, this paper sheds initial light on the crosstalk between macrophages and endothelial cells.

In the same direction, Löffler et al. showed that disturbed bone regeneration in osteotomized aged rats was associated with impaired M2 macrophage function and consequent reduced revascularization of the bone callus. Accordingly, local infusion of CD14^+^ macrophage precursors into the osteotomy gap of aged rats reduced fibrosis and improved vascularization and overall bone regeneration. This work paves the way to more extensive studies of macrophage dynamics in early phases of bone healing, in relation to outcome.

## Mast Cells in Bone Homeostasis

Mast cells (MCs), commonly referred to as tissue-resident immune cells promoting allergic reactions, have been demonstrated to also be involved in the pathophysiology of bone. Their effect is essentially elicited through the release of the content of secretory granules, that is, soluble mediators, including histamine, heparin, cytokines, growth factors and enzymes, which influence bone metabolism, potentiating either osteoclast or osteoblast activity. In this respect, Ragipoglu et al. provided a critical overview of the current knowledge of MC function in bone homeostasis and disease, specifically focusing on osteoporosis and bone regeneration. Owing to the lack of a relevant human condition lacking MCs, most of the literature has been derived from experimental murine models, and was sometimes contradictory. Despite the need for further investigation, the proposal to exploit MC-targeting drugs in the framework of bone diseases constitutes an attractive option.

## Soluble Factors on Stage

In addition to the prototypical RANKL/RANK axis, other signaling pathways, like Wnt signaling, also have a clear osteoimmunological relevance. Goes et al. investigated this pathway in the context of periodontitis, an infectious disease of the alveolar bone and surrounding tissue in which an exacerbated inflammatory host response to an oral biofilm causes massive tissue destruction. In particular, the authors focused on the contribution of the osteocyte-derived Dkk1 molecule, a secreted inhibitor of the Wnt signaling induced by inflammatory mediators in the periodontal tissue, to disease progression. They found that in a model of experimental periodontitis, osteocyte-specific Dkk1 deletion dampened bone loss by acting both on osteoblast and on osteoclast parameters and limiting inflammatory infiltrates. This result underlined the role of the local milieu in determining periodontal bone regulation. Further investigation is required to clarify the immunomodulatory properties of Dkk1 and its possible role as a target in inflammatory bone loss conditions.

In the molecular crosstalk between bone and immune cells, an important role is played by chemokines, a large family of ligands (and corresponding receptors) commonly known to direct homing of immune cells, development, and inflammation. In addition to these functions, autocrine and paracrine chemokine signaling in the bone tissue regulate osteoblast and osteoclast functions in pathophysiological conditions. Brylka and Schinke reviewed the current knowledge on this topic, with major emphasis on the most established subsets of chemokines, for example, CCL2, CCL3, CCL20 and the CXCL12/CXCR4 axis. As envisaged by the authors, the scrutiny of ill-defined aspects of chemokine biology in the framework of bone metabolism can be clinically relevant, based on the pleiotropism of these molecules.

Similar considerations apply to the prototypical long pentraxin PTX3, mostly known for its role in innate immunity, inflammation and matrix remodeling, and recently emerging as an active player in bone pathophysiology. Parente et al. provided an overview of the novel *in vitro* and *in vivo* findings pointing to the role of PTX3 in stimulating osteogenic function. By contrast, evidence in humans and in experimental models suggests PTX3 may have pro-osteoclastogenic effects, particularly in inflammatory conditions and skeletal chronic diseases. The structural complexity of this molecule would indeed allow a wide range of (likely context-dependent) interactions, whose exploitation for specific therapeutic purposes could be of interest and foster research.

## Glucocorticoids in Osteoimmunology

Endogenous glucocorticoids (GCs) represent a paramount stress response mechanism in the body. Based on their established immunomodulatory effect, GC are also abundantly exploited as drugs in different conditions. In addition, GC exert modulatory effects in diverse other contexts: Ahmad et al. reviewed direct and indirect effects of GC on bone and immune cells and on their crosstalk with each other and with vasculature and muscle. The authors paid specific attention to GC action in osteoporosis, inflammatory bone diseases and bone regeneration, and underlined the need for a more holistic approach in including all the players in the same picture.

## The Environmental Clue

Bone tissue engineering translates the osteoimmunological principles into practice to face a growing medical need, particularly when considering the huge number of bone grafts implanted annually worldwide. Immunomodulatory properties are inherent to many components of endogenous extracellular matrices (ECM), including collagen fibers, hyaluronans, and heparin sulfate. García-García and Martin illustrated how material properties can be designed *ad hoc* for different purposes. In particular, they highlighted a new generation of biomaterials, that is, immunoinstructive ECM, able to direct the host immune cell behavior and to guide the spatiotemporal release of endogenous immunoregulators promoting efficient bone repair.

## Conclusion

At variance with the old-fashioned concept of bone as inert material with pure mechanical functions, the current view depicts the skeleton as a lively tissue actively interacting with all other tissues and organs in the body. In parallel, the innate immunity arm is now an established player with physiological relevance in bone homeostasis. A more thorough understanding of the interaction modes between these cell types and molecular cues might effectively impact on large population groups, thus warranting current and future efforts.

## Author Contributions

All authors listed have made a substantial, direct and intellectual contribution to the work, and approved it for publication.

### Conflict of Interest

The authors declare that the research was conducted in the absence of any commercial or financial relationships that could be construed as a potential conflict of interest.
